# Religious coping strategies for people with HIV/AIDS (PLWHA) Muslims in Indonesia: A qualitative study with a telling-the-stories

**DOI:** 10.1016/j.heliyon.2022.e12208

**Published:** 2022-12-19

**Authors:** Baidi Bukhori, Ema Hidayanti, Dominikus David Biondi Situmorang

**Affiliations:** a Department of Psychology, Faculty of Psychology and Health, Universitas Islam Negeri Walisongo Semarang, Jl. Walisongo No. 3-5, Semarang, Jawa Tengah 50185, Indonesia; b Department of Islamic Guidance and Counseling, Faculty of Da’wah and Communication, Universitas Islam Negeri Walisongo Semarang, Jl. Walisongo No. 3-5, Semarang, Jawa Tengah 50185, Indonesia; c Department of Guidance and Counseling, Faculty of Education and Language, Atma Jaya Catholic University of Indonesia, Jl. Jenderal Sudirman 51, DKI Jakarta 12930, Indonesia

**Keywords:** Religious coping strategies, Psychosocial-spiritual problems, PLWHA, Qualitative study

## Abstract

**Objective:**

The purpose of this study is to find out more about the psycho-social-spiritual problems experienced by People with HIV/AIDS (PLWHA) Muslims and their efforts to overcome them by using religious coping.

**Methods:**

This research is a qualitative research method with a telling-the-stories approach. This study describes assumptions about the physical/behavioral, social/emotional, cultural/historical, and spiritual aspects related to clinical participants' body, life, and power. In the context of this research, telling the stories from HIV/AIDS patients about how psycho-social-spiritual problems are experienced and efforts to overcome them with religious coping. This study involved 33 HIV/AIDS patients informants at Central General Hospital (RSUP) of Dr. Kariadi Semarang, Central Java with the criteria of being Muslim, medication adherence (ARV therapy).

**Findings:**

The results showed that most PLWHA experienced physical complaints such as pain in the early days of taking ARVs, opportunistic infections such as Stevens-Johnson, dizziness, temporary blindness, and body stiffness. Psychological problems including stress, anxiety, fear of death, and guilt. The physical and psychological problems experienced by PLWHA encourage them to use religious coping such as praying, dhikr, and prayer. This religious coping has a calming effect, which impacts reducing physical complaints and overcoming psychological problems. The psychoneuroimmunology pathway can explain the physical and psychological relationship, which shows that favorable psychological conditions trigger the nerves to work optimally to increase immunity.

**Discussion:**

In conclusion, religious coping can be used to overcome the bio-psycho-social-religious problems of PLWHA. This strengthens the application of holistic therapy to PLWHA through palliative care to handle pain and other physical complaints and psychosocial-spiritual concerns.

## 1 Introduction

The increasing number of cases of HIV/AIDS from year to year demands serious attention to efforts to deal with it. This has prompted the issuance of the Decree of the Minister of Health of the Republic of Indonesia Number HK.01.07/MENKES/90/2019 concerning the National Guidelines for HIV Treatment Medical Services (Keputusan Menteri Kesehatan, 2019). These guidelines become a national reference in managing People with HIV/AIDS (PLWHA), from HIV detection and ARV therapy to managing opportunistic infections and comorbidities in PLWHA. The existence of this national guideline certainly makes it easier to handle PLWHA who have complex problems (bio-psycho-socio-religious). One of the physical problems that PLWHA often experience is pain. Parker et al. (2014) stated that 55%–67% of the pain is present at every stage of the development of HIV/AIDS. Coughlan (2003) explained that pain in PLWHA is caused, among others, by the virus itself, opportunistic infections, drugs that treat several antiretroviral agents, debilitating effects of disease, malnutrition, meningitis, herpes, and respiratory infections (tuberculosis).

Pain in PLWHA is associated with the causes above. However, it is often related to psychological or psychiatric symptoms that accompany or are experienced by PLWHA. Several studies have shown this, including Scott et al. (2018) states that pain is associated with psychosocial factors such as depression, psychological distress, post-traumatic stress, drug abuse, sleep disturbances, decreased ARV adherence, use of health care, interrupted VCT clinic visits, unemployment, and psychological protection factors. Merlin et al. (2012) also revealed that patients who have 40% more psychological or psychiatric symptoms experience pain than patients who do not have psychological symptoms. They further explained that the psychological symptoms were worry, feeling sad, having trouble sleeping, feeling irritable, nervous, and having difficulty concentrating. Newshan et al. (2002) amplify pain in PLWHA accompanied by other symptoms such as restlessness, fatigue, and difficulty sleeping, which affect the quality of life.

Physical problems in PLWHA that are triggered by psychological issues make the situation of PLWHA increasingly complex. This is considering that one of the causes of stress or psychosocial stressors is physical illness (Mustamir, 2010). HIV/AIDS, which is included in the category of terminal illness, is a source of stress for sufferers. As stated by Deekshitulu (2015), that initial stress causes depression. This is a mental condition commonly experienced by PLWHA. Depression in HIV/AIDS patients is a state of low mood and aversion to activities that can affect a person’s thoughts, behavior, feelings, and sense of well-being. Depressed people can feel sad, anxious, empty, hopeless, worried, helpless, and worthless (Deekshitulu, 2015).

Seeing the circle of the relationship between the bio-psycho-socio-spiritual problems of PLWHA above, essentially every PLWHA must have the ability to deal with these problems properly. Therefore, it is interesting to explore more about the experiences of PLWHA Muslims in utilizing religious coping to deal with various issues faced by being PLWHA. This qualitative study took the object of Muslim HIV/AIDS patients at the Central General Hospital (RSUP) of Dr. Kariadi Semarang, Central Java. Exploring the experiences of PLWHA Muslims related to religious coping is becoming increasingly interesting as there is a significant finding that the majority of HIV/AIDS patients at the Central General Hospital (RSUP) of Dr. Kariadi Semarang, Central Java, became more religious after being PLWHA (Hidayanti, 2020). Central General Hospital (RSUP) of Dr. Kariadi Semarang was chosen as the research location because it was the first referral hospital for people living with HIV/AIDS (PLWHA) in Central Java since 2006 (Keputusan Menteri Kesehatan, 2006). This hospital is a referral place for PLWHA who are not treated at a referral hospital for PLWHA at the district/city level.

Furthermore, the context of the discussion in this study will be explained, that the term in psychological concepts is often referred to as coping strategies, namely various mental and behavioral efforts to master, tolerate, reduce, or minimize a stressful situation or event. Lua et al. (2012) mentioned four: Emotion-Focused Coping (EFC), Behavioral-Coping (BC), Cognitive Appraisal (CA), and Problem-Focused Coping (PFC). While Lazarus and Folkman (1984) simplify coping, there are two, namely, first, EFC is a form of coping directed to regulate emotional responses to stressful situations. Individuals can control their emotional reactions with behavioral and cognitive approaches. The second is PFC, a form of coping that is more directed at efforts to reduce the demands of stressful situations, meaning that coping that appears is focused on individual problems in dealing with stress by learning new ways or skills.

Coping that involves religion is called by Trevino et al. (2010) as a Religious Coping Strategy, which is included in Emotional-Focused Coping. Religious Coping Strategy is a coping strategy that involves religion in problem-solving by increasing religious rituals. This type of coping is a variety of efforts made by individuals applying religious elements to regulate or overcome differences between internal and external demands to help them overcome stress (Becker et al., 2006). This means that each individual can develop their coping strategies based on the observance of their religious teachings. This is in line with Tiliouine and Belgoumidi (2009), who interpret religiosity as an expression of practice and behavior that originates from the religion they adhere to. Religious coping strategies are expressions of practice and behavior that come from their religion when dealing with problems (Daulay et al., 2022).

Departing from this opinion, PLWHA Muslims express practices and behaviors that come from religion when dealing with complex problems (bio-psycho-socio-spiritual). This is reinforced by the theory of Padela and Zaganjor (2014) regarding The Psychological Measure of Islamic Religiousness (PMIR), which makes positive religious coping and identification methods one of the important dimensions in measuring Muslim religiosity, in addition to the dimensions of punishing Allah reappraisal, and the Islamic ethical principles and universality. The positive dimension of religious coping and identification methods measures the extent to which a person uses positive religious coping methods (reading the Qur’an, asking for forgiveness, growing trust in God) to deal with life’s stressors and build their intrinsic motivation in worship (Padela and Zaganjor, 2014). Following this theory, it becomes interesting to explore further the experiences of PLWHA Muslims in utilizing religious coping to deal with various problems faced by being PLWHA. Furthermore, the purpose of this study is to find out more about the bio-psycho-socio-spiritual problems experienced by PLWHA Muslims and their efforts to overcome them by using religious coping, this is a continuation of previous research (Bukhori et al., 2021).

## 2 Methods

This research is a qualitative research method with a telling-the-stories approach. Miller and Crabtree (1994) suggest that qualitative research in the clinical field can take advantage of methodologically convincing life stories or rhetorically convincing stories. This study describes assumptions about the physical/behavioral, social/emotional, cultural/historical, and spiritual aspects related to clinical participants' body, life, and power. In the context of this research, telling-the-stories from HIV/AIDS patients about how bio-psycho-socio-spiritual problems are experienced and efforts to overcome them with religious coping. This study involved 33 HIV/AIDS patients informants at Central General Hospital (RSUP) of Dr. Kariadi Semarang, Central Java with the criteria of being Muslim, medication adherence (ARV therapy).

Data collection techniques are in-depth interviews or interviews with patients, doctors, and peer assistants. The validity of the data applying the member checking technique is to bring back the final report or descriptions or specific themes in front of the participants to check whether the description is accurate, according to the data provided by the informant. At the same time, the data analysis technique uses qualitative analysis with a narrative strategy emphasizing research that involves retelling participants' stories using structural elements such as plot, setting, activity, climax, and ending. In this context, PLWHA tells various bio-psycho-social-spiritual problems and how to handle them by involving teachings and worship following the Islamic religion that they believe in.

The authors identified 8 PLWH whose statements were chosen to represent the whole. This determination is based on the similarity of answers that are most often expressed by the patients. The answers summarized are their assumptions about the physical/behavioral, social/emotional, cultural/historical, and spiritual aspects related to clinical participants' body, life, and power. This is done so that the subjectivity of authors in leading research results related to the credibility and validity of the findings can be guaranteed.

All procedures involving human participants in this study complied with the institutional and/or national research committee’s ethical standards (the Code of Professional Ethics for Psychologist and Psychology scientist, Indonesian Psychological Association [HIMPSI]), as well as the 1964 Helsinki declaration and its subsequent amendments or comparable ethical standards. Furthermore, informed consent was obtained from all participants for this study.

## 3 Findings

Religious coping will be presented in two sub-chapters, namely religious coping (expression of practice and behavior that comes from religion) when dealing with physical complaints or problems and religious coping (expression of practice and behavior originating from religion) when facing bio-psycho-socio-spiritual problems. Religious coping strategies here will describe the feelings experienced by patients when they are diagnosed with HIV/AIDS and other accompanying psychological problems, various types of positive religious coping, and the benefits of applying positive religious coping. Positive religious coping means using spiritual teachings in attitudes and behavior or worship in dealing with problems.

### 3.1 Religious coping for handling the physical problems of PLWHA muslims

HIV/AIDS patients who experience physical problems such as leg pain, painful swallowing, herpes skin problems, and others due to the effects of drugs, viruses, and opportunistic infections state that they do regular consultations and check-ups with doctors to get drugs. In addition, they also try to develop their own coping to overcome the physical problems that they experience. Pain conditions or other physical complaints that are often experienced do not entirely rely on the drugs given by the doctor. Even one patient who often experiences headaches and feels uneasy when consulting a doctor is advised to do things that make him relaxed and relaxed. The choice made by AN (25-year-old) was to listen to the murottal Al-Qur’an, as stated below:

*“Lately I’m often not calm, my head hurts, the doctor is advised to do activities that can make me relax, I choose to listen to the murotal al-Qur’an, yes I feel… I started to calm down, the pain in my head was also somewhat reduced, and I could sleep. When I relax, the pain becomes less and disappears.”*

The patient’s statement above was confirmed by one of the doctors at the Infectious Disease Clinic, Dr. Kariadi Semarang. According to him, HIV/AIDS patients are always motivated to get closer to God to be healthier, free from TB and other infections. A TR patient (36-year-old) confirmed that the doctor motivated her to pray, even gave her a mukena when she was undergoing treatment. A TR admits to being calmer even though she has not entirely performed her five daily prayers. HIV/AIDS patients need peace of mind, primarily in-depth interviews. It was found that the patients experienced physical complaints and had psychosocial problems with their families.

*“At that time, I was hospitalized for a month here. It felt like I couldn’t do anything; my body felt weak. I never thought I would experience pain like this. I had no idea why my husband could have a naughty girlfriend; that’s where I got infected. The one who has been taking care of me is Doctor Muchlis; he is a very nice person. When he examined me, he asked if I had prayed or not; then, when he came back, I was brought a mukena. Thank God, now I’m healthy again. I surrender sincerely and pray a lot. I still want to be healthy and be able to watch the children grow up”* (Interview with TR).

AG did the same thing, who began to routinely tahajjud after being PLWHA. For AG, the most important thing was to organize one’s heart after being a PLWHA, so that it was suggested to do the tahajjud prayer. Moreover, AG did not only experience the physical effects of ARVs, but also fear anxiety, and feelings of guilt. It is narrated as follows:

*“I’m lucky because I’m still healthy, even if I go anywhere I still don’t feel confident, sometimes I think I can’t be like others, can I get married and have children. I feel lucky because my mother understands very well; she asked me not to think about things. The important thing is to be healthy, pray diligently. Unlike before when living in Surabaya, all prayers must be abandoned. After being hit by this virus, I started diligently praying tahajjud to be calmer. But it’s true; I feel better, I used to be afraid until I was just afraid to sleep until in the end I liked being looked after by Mother”* (Interview with AG).

Worship is not the only way to deal with patients' pain or other physical problems. Patients who experience physical problems due to drug infections admit that they are limited in performing worship, including prayer. Nevertheless, they have faith that they will be healthy again and pray for healing from Allah SWT. This experience was shared by TR, RY, and AN, who had opportunistic infections and underwent long treatment for recovery. The three of them acknowledged their belief and surrendered to God’s help that allowed them to survive through the critical period at that time. Peer mentors confirmed that optimism and enthusiasm for life helped patients whose physical condition worsened. If this is not owned, the patient will give up and eventually die like the last three patients who were accompanied.

YN (42-year-old) explained that closeness to Allah and accepting his pain was key to surviving. This is as stated below:

*“My condition is challenging, I have to continue ARV therapy, but as a result, there is swelling in the neck and must be operated on. It doesn’t just feel painful, yes, but it hurts and must be taken as well, like me, who does nothing but get HIV if you don’t ask God to pray, recite the Koran, dhikr, you’re desperate, you’re tired like this all the time.”*

Thus, patients develop non-pharmacological management in dealing with physical complaints that are felt. Non-pharmacological management by developing religious coping utilizes worship and belief in Allah to help deal with pain and other physical complaints. This provides its strength for patients to present different experiences to reduce the perceived complaints. In addition, it is known that the patient understands that unfavorable social-psychological conditions also trigger the physical complaints that are felt. The choice of religious coping is also a means to calm the soul that is afraid, worried, anxious, and stressed due to other problems that accompany being PLWHA (Hidayanti, 2013; Hidayanti and Syukur, 2018).

### 3.2 Religious coping for handling psychosocial-spiritual problems with PLWHA Muslims

The psychological dynamics of sadness, shock, anger, and disappointment experienced by housewives PLWHA from their husbands were responded very positively by each informant. Almost all of them emphasized that the events they encountered were the destiny of Allah SWT that must be accepted and lived. Some informants admitted that it was challenging to accept the reality at first. Still, the solution was to return all matters to Allah SWT, as stated by informants below:

*“God’s destiny has been outlined, but it depends on our behavior. I am not my destiny; praying becomes peaceful, fasting has an effect in that direction... I also fast Monday and Thursday; moreover, praying must be closer toGod...If you do a lot of worship, your sins will be melted away; it hurts to dissolve your sins, right? I hope so too… Getting closer to God with prayer reduces guilt, sins, makes you feel calmer, makes you have a high spirit of life…”* (Interview with G-10).

Developing positive religious coping strategies were also taken by G-9 informants to overcome feelings of sadness and fear of death:

*“Afraid to die too, hahaha (patient laughs). Worship is more time when there are a lot of problems, especially when it’s like this. If you die, it’s good just to die, but I’m afraid what my sins will be”* (Interview with G-9).

*“Being close to God gives you a direction in life; life is more meaningful… Dizzy from work, finally praying, there is a way out. Optimistic in treatment, because God who gave this means that God will also heal. Medicine is only a means, so you are more afraid if you want to sin. My Christian boyfriend often goes to church; since he is positive, he is afraid he won’t go to church again. Currently, I am happier; the grace of sustenance, health, and work come from Allah”* (Interview with G-5).

The statement above confirms that worship helps them find a better meaning in life, makes it easier to find a way out of difficulties, is more alert in their actions, and motivates them to seek treatment diligently.

## 4 Discussion

### 4.1 Religious coping for handling the physical problems of PLWHA muslims

Sabin et al. (2018) stated that pain symptoms are generally faced by PLWHA even though ARV therapy has been declared effective, which affects the patient’s quality of life. Based on this statement, it can be justified that some patients who routinely undergo ARV therapy do not rule out the possibility of still experiencing pain. The source of pain in the patient is known to be very religious. This is reasonable because it is said by Mccollum and Pittman (2010) that the source and location of pain in PLWHA can spread from the head, chest, stomach, mouth, feet, skin, herpes, and rheumatic pain. Based on the study results, it is known that each patient has various sources and locations of pain, leg pain, headache, and stomach pain are generally new to patients who are adapting to ARV drugs. In contrast, oral pain is difficult to swallow due to opportunistic infections (AN, RY, TR).

The causes of pain experienced by patients also differ from the effects of treatment due to viruses and opportunistic infections. This is by the opinion of Coughlan (2003) which states that pain in HIV patients is caused, among others, by the virus itself, opportunistic, debilitating infections, malnutrition, meningitis, herpes, and respiratory infections (tuberculosis), side effects of drugs, specific manifestations of the disease. End-stage disease and other non-HIV-related causes. In addition to knowing the physical source of pain, various psychosocial factors that contribute to the pain that arise are also found. The results show that in general, patients who experience physical pain are also faced with psychological problems such as worry, anxiety, stress, feeling guilty, irritated, and angry. Psychological factors can indeed trigger the emergence of pain in HIV/AIDS patients, as mentioned by Merlin et al. (2012), Newshan et al. (2002), and Rotheram-Borus (2000). Psychological factors include emotional stress, depression, anxiety, worry, anxiety, and trauma.

On the other hand, it was also found that patients were faced with social problems such as separation from their partner, loss of children, and lack of support from family. McCollum and Pittman (2010) referred to this pain trigger factor as a life stressor factor and family and community attitudes. Pain triggers in this factor including financial stress, death in the family, opportunities to access housing and good health services, resistance to disease, social support, and attention to treatment. From this, it is known that PLWHA who experience pain are caused by many factors, not only due to a decrease in treatment adherence (Scott et al., 2018), but also due to the loss of social support from partners as experienced by TR and AN. Perez et al. (2008) also confirmed a significant relationship between spiritual, social support, and pain experienced by HIV/AIDS patients. Loss of social support can adversely affect the psychological and physical conditions of PLWHA (Sofro and Hidayanti, 2019).

The experience of HIV/AIDS patients in this study showed that the religious coping developed began by increasing the intensity of worship such as praying, reading the Qur’an, listening to the Qur’an, and dhikr. In addition, religious coping is also a positive attitude towards the pain that is felt. Chen et al. (2020) confirm that coping strategies play an essential role in maintaining mental health in HIV/AIDS patients in China; the right coping skills lead to a long life for PLWHA. Religious coping is one type of coping that HIV/AIDS patients can use in dealing with physical and psychosocial problems. Ironson et al. (2020) proved that religious coping could increase the CD4 count of HIV/AIDS patients and undetectable Viral Load. This strengthens the importance of religious coping carried out by PLWHA in dealing with pain and physical disorders that they experience.

The patient recognizes religious coping as presenting a comfortable and relaxed condition that can calm an anxious soul or heart (Mar’ati and Chaer, 2017), can also foster hope and trust (submission to Allah), which can reduce stress (Husnar et al., 2017). A good psychological condition can ultimately reduce the symptoms of pain that are felt. Addington et al. (2020), emphasize positive skills in reducing pain disorders and preventing an increase in opioid analgesics (pain medication). Pain management with opioids and morphine is controversial, highly regulated, expensive, and has long-term impacts (Coughlan, 2003; Ownby, 2006). This, of course, requires developing positive skills in dealing with non-pharmacological pain. Various non-pharmacological therapies for pain management that have been developed include dry cupping (Subadi, 2014), lemongrass aromatherapy (Putri et al., 2019), music therapy (Situmorang, 2021a, 2022; Situmorang et al., 2018), dance therapy (Situmorang, 2021b), rapid counseling (Situmorang, 2021c, 2021d), warm ginger compresses (Pujastuti et al., 2018), acupuncture therapy (Yulianto, 2009), ginger therapy (Rahmawati et al., 2018), yoga exercises (Devi et al., 2019), Islamic guidance and counseling (Aristiana et al., 2017), and ice massage (Ownby, 2006).

### 4.2 Religious coping for handling psychosocial-spiritual problems with PLWHA Muslims

Prayers 5 times in the first place as a method that is widely used by informants. This is directly proportional to the large number of informants who are increasingly diligent in praying five times after being diagnosed with HIV/AIDS. This reality confirms that the intensity of worship encourages informants to feel the benefits of every worship performed. One of the benefits most felt by most informants in all categories is peace of mind by praying five times a day. This can be observed from the previous G-10 and G-5 informants' confessions.

Some of the confessions above show that prayer is used as a means for them to complain about the problems that they face. This brings relief and peace of mind. Every troubled individual is encouraged to vent the problems he/she faces. This constructive channeling of emotions is known as catharsis. The release of suppressed emotions can be a beneficial therapeutic effect (Wahyuningsih, 2017). Prayer can be used as an effective and constructive means of self-catharsis when facing problems, moreover, they are still limited to opening up the status of PLWHA, so they cannot complain about just anyone.

Self-catharsis in prayer is a form of communication of a servant to his/her Lord. Complaining, complaining, venting (pouring out) is an integral part of a series of prayers said to Allah SWT. The dialogue can be likened to counseling. As emphasized by Moriarty and Hoffman (2014), this states that God is considered an absent counselor, a counselor who is not present but can listen to all stories and can feel His power as the Highest. So it can be understood that the prayers performed as a means for informants to complain about problems and difficulties in life, in the end, bring peace of mind.

The peace of heartfelt by the informants by praying is a positive religious coping because there is a series of prayers in the prayer itself. Sangkan et al. (2005) argue that prayer can reduce anxiety because it includes regular prayer, relaxation (prayer movements), hetero or autosuggestions in prayer readings, therapy (me and Allah), and hydrotherapy. The prayer contains a psychotherapeutic element because it contains spiritual/spiritual power that evokes self-confidence and a sense of optimism (hope for healing). These two things are essential for the healing process of disease in addition to drugs and medical actions (Hawari, 2000).

Prayer as a form of remembrance (remembrance of Allah) can lead to inner peace as referred to in the Qur’an letter Ar-Ra'd' verse 28: *Those who believe and their hearts are at peace with the remembrance of Allah. Remember it is only by remembering Allah that the heart becomes at peace'*. Syukur (2012) asserts that inner peace arises because remembering Allah encourages the human brain to melt happiness chemicals called endorphins. Pearce et al. (2015) and Situmorang (2021e) mention that religious cognitive behavioral therapy has a positive effect on endocrine function (regulating stress hormones such as cortisol). Thus it can be understood that informants who perform prayers (prayers and remembrance of Allah) can feel psychological therapy such as reducing/eliminating anger, sadness, anxiety, anxiety, fear, and stress. Prayer is not only the removal of vile and evil deeds but the removal of sorrow and distress and the cause of the elimination of sins (Az-Zuhaili, 2009).

The disappearance of the various psychological problems above creates all informants' peace. Moreover, the informants who were infected due to risky behavior admitted that praying and remembrance were done to eliminate the fear of death and reduce feelings of sin. Confession of one of the MSM category informants: *“At first, I felt unfortunate… I cried when I called my partner… Yes, now we can finally support each other, because both of them have been hit… Afraid to die too, hahaha… (patient laughs)*. *Worship is more timely if there are many problems, especially like this…. If you die, it’s good just to die, but I’m afraid what will happen to my sins*” (Interview with G-9).

Psychotherapeutic effects can give birth to positive psychology needed to fight against the HIV/AIDS virus. Positive psychology in Islamic teachings can be in patience, trust, and sincerity in living life with HIV/AIDS. The positive attitudes and behaviors come from Islamic teachings that the informants own reference to an adaptive spiritual response. Nursalam quoting that adaptive spiritual responses emphasize the patient’s acceptance of the illness he is suffering so that HIV patients can sincerely accept their disease and take lessons. The adaptive spiritual response in HIV/AIDS patients consists of 3 things: strengthening realistic expectations for healing, taking lessons, and grit.

## 5 Conclusions

The results of this study indicate that HIV/AIDS patients generally experience subjective pain. Various sources of physical pain experienced by patients also found psychosocial and religious factors that contribute to the pain. Psychosocial and spiritual problems that accompany the pain including stress, anxiety about the future, fear of death, feelings of guilt, and guilt. Proper pain management needs to be done not only pharmacologically but also non-pharmacologically. Religious coping is an alternative option that can be developed in non-pharmacological pain management to implement a biopsychosocial model and palliative therapy. Religious coping as a medium to deal with psychosocial and spiritual problems that affect pain/other physical disorders. The reduction in the patient’s psychosocial-spiritual problems can affect the pain felt with the psychoneuroimmunology (PNI) approach, which explains that positive psychological conditions can increase individual immunity. The results of this study increasingly show the importance of spiritual support in managing pain in PLWHA, in addition to pharmaceutical therapy. In addition, it shows that religious coping is beneficial for PLWHA to overcome their problems. These findings further strengthen the importance of implementing holistic treatment for PLWHA through palliative care to manage pain and other physical complaints and patients' psychosocial-spiritual issues.

## 6 Limitations

This study only focuses on PLWHA Muslims, so the results of this study cannot provide a broader picture of the religious coping strategies experienced by PLWHA from other religions. In addition, this study has not identified further related to emotional-focused and problem-focused coping skills possessed by PLWHA Muslims, so this can enrich the following findings.

## 7 Suggestions

It is recommended that further research be conducted on the religious coping strategies experienced by PLWHA from other religions, such as Catholicism, Protestantism, Buddhism, Hinduism, Confucianism, and others. In order to get a more comprehensive finding, this can be an interesting topic to do a comparative study. In addition, it is also hoped that PLWHA will be able to provide tests on emotional-focused and problem-focused coping skills for all religions, in order to get a more comprehensive picture.

## 8 Implications

The results of this study have significant ramifications for how religious coping mechanisms are seen in hospital settings, particularly for future mental health professionals. By utilizing the findings of this study, policy makers in hospitals, rehabilitation centers, and counseling agencies are expected to continue to pay attention to this matter, so as to improve their religious coping strategies.

## 9 Future research directions

Future research that is more in-depth can be guided by the results of this study. It is very important to choose a future research program on this subject in a hospital. In addition, there is still no specific solution to intervene for those who have this problem of poor religious coping strategies that have been proven to be efficient in clinical and pastoral care. Therefore, it is crucial to undertake more study to ascertain the type of intervention that will be most successful in resolving this issue.

## Declarations

### Author contribution statement

Baidi Bukhori; Ema Hidayanti: Conceived and designed the experiments; Performed the experiments; Wrote the paper.

Dominikus David Biondi Situmorang: Analyzed and interpreted the data; Contributed reagents, materials, analysis tools or data; Wrote the paper.

### Funding statement

This research did not receive any specific grant from funding agencies in the public, commercial, or not-for-profit sectors.

### Data availability statement

Data will be made available on request.

### Declaration of interest’s statement

The authors declare no conflict of interest.

### Additional information

No additional information is available for this paper.
